# The use of microbead-based spoligotyping for *Mycobacterium tuberculosis *complex to evaluate the quality of the conventional method: Providing guidelines for Quality Assurance when working on membranes

**DOI:** 10.1186/1471-2334-11-110

**Published:** 2011-04-28

**Authors:** Edgar Abadia, Jian Zhang, Viviana Ritacco, Kristin Kremer, Raymond Ruimy, Leen Rigouts, Harrison Magdinier Gomes, Atiná Ribeiro Elias, Maryse Fauville-Dufaux, Karolien Stoffels, Voahangy Rasolofo-Razanamparany, Darío Garcia de Viedma, Marta Herranz, Sahal Al-Hajoj, Nalin Rastogi, Carlo Garzelli, Enrico Tortoli, Philip N Suffys, Dick van Soolingen, Guislaine Refrégier, Christophe Sola

**Affiliations:** 1 Institute of Genetics and Microbiology UMR8621, CNRS Université Paris-Sud 11 Universud, Campus d'Orsay, F-91405 Orsay-Cedex, France; 2 Instituto Nacional de Enfermedades Infecciosas ANLIS Carlos Malbrán, Vélez Sarsfield 563, 1281 Buenos Aires, Argentina; 3 National Institute for Public Health and the Environment, Bilthoven, The Netherlands; 4 EA 3964 Université Paris-Diderot & Microbiology Laboratory, Bichat-Claude Bernard Hospital AP-HP, Paris, France; 5 Mycobacteriology Unit, Prince Leopold Institute of Tropical Medicine, 155 National Straat, 200 Antwerp, Belgium; 6 Laboratory of Molecular Biology applied to Mycobacteria, Oswaldo Cruz Institute, Rio de Janeiro, Brazil; 7 National Reference Centre of Tuberculosis and Mycobacteria, Scientific Institute of Public Health, Brussels, Belgium; 8 Unité des Mycobactéries, Institut Pasteur de Madagascar, Antananarivo, Madagascar; 9 Servicio de Microbiología Clínica y Enfermedades Infecciosas, Hospital Gregorio Marañón, Madrid, Spain; 10 CIBER Enfermedades Respiratorias (CIBERES), Spain; 11 Department of Comparative Medicine, King Faisal Specialist Hospital and Research Center, Riyadh, Saudi Arabia; 12 Unité de la Tuberculose et des Mycobactéries - WHO Supranational TB Reference Laboratory, Institut Pasteur de Guadeloupe, Abymes, Guadeloupe; 13 Dipartimento di Patologia Sperimentale Biotecnologie Mediche Infettivologia ed Epidemiologia, Università di Pisa, I-56127 Pisa, Italy; 14 Regional Reference Center for Mycobacteria, Careggi Hospital, viale Morgagni 85, 50134 Firenze, Italy; 15 Department of Pulmonary Diseases and Department of Microbiology, Radboud University Nijmegen, P.O. Box 9101, 6500 HB Nijmegen, The Netherlands; 16 Unité de Génétique Mycobactérienne, Institut Pasteur, 25-28 rue du Dr. Roux, F-75724 Paris-Cedex 15, France

## Abstract

**Background:**

The classical spoligotyping technique, relying on membrane reverse line-blot hybridization of the spacers of the *Mycobacterium tuberculosis *CRISPR locus, is used world-wide (598 references in Pubmed on April 8th, 2011). However, until now no inter-laboratory quality control study had been undertaken to validate this technique. We analyzed the quality of membrane-based spoligotyping by comparing it to the recently introduced and highly robust microbead-based spoligotyping. Nine hundred and twenty-seven isolates were analyzed totaling 39,861 data points. Samples were received from 11 international laboratories with a worldwide distribution.

**Methods:**

The high-throughput microbead-based Spoligotyping was performed on CTAB and thermolyzate DNA extracted from isolated *Mycobacterium tuberculosis *complex (MTC) strains coming from the genotyping participating centers. Information regarding how the classical Spoligotyping method was performed by center was available. Genotype discriminatory analyses were carried out by comparing the spoligotypes obtained by both methods. The non parametric U-Mann Whitney homogeneity test and the Spearman rank correlation test were performed to validate the observed results.

**Results:**

Seven out of the 11 laboratories (63 %), perfectly typed more than 90% of isolates, 3 scored between 80-90% and a single center was under 80% reaching 51% concordance only. However, this was mainly due to discordance in a single spacer, likely having a non-functional probe on the membrane used. The centers using thermolyzate DNA performed as well as centers using the more extended CTAB extraction procedure. Few centers shared the same problematic spacers and these problematic spacers were scattered over the whole CRISPR locus (Mostly spacers 15, 14, 18, 37, 39, 40).

**Conclusions:**

We confirm that classical spoligotyping is a robust method with generally a high reliability in most centers. The applied DNA extraction procedure (CTAB or thermolyzate) did not affect the results in this study. However performance was center-dependent, suggesting that training is a key component in quality assurance of spoligotyping. Overall, no particular spacer yielded a higher degree of deviating results, suggesting that errors occur randomly either in the process of re-using membranes, or during the reading of the results and transferring of data from the film to a digital file. Last, the performance of the microbead-based method was excellent as previously shown by Cowan *et al*. (J. Clin. Microbiol. 2004) and Zhang *et al*. (J. Med. Microbiol. 2009) and demonstrated the proper detection of spacer 15 that is known to occasionally give weak signals in the classical spoligotyping.

## Background

Clustered Regularly Interspaced Palindromic Repeats (CRISPRs) are a family of DNA repeats of 21 to 37 bp that are separated by regularly sized, non repetitive unique DNA spacer sequences [1]. CRISPRs are present in the genomes of most bacteria (40%) and archaea (90%) [2]. It is believed that some spacers originate from mobile genetic elements [1] and it has been shown that they confer ''immunity'' against bacteriophages and plasmids [2-4].

Studies on the CRISPR region of *Mycobacterium tuberculosis *complex (MTC) strains started in 1993 [5]. In 1995, spoligotyping was for the first time mentioned in the article describing the Beijing genotype of *Mycobacterium tuberculosis *[6]. In 1997, Kamerbeek *et al*. provided a standardized reverse line blot hybridization method (*sp*acer *oligo*nucleotide *typing *= spoligotyping) to genotype MTC complex strains based on polymorphism of this region, and soon after, corresponding membranes were commercialized [7,8]. Since then, numerous studies have used spoligotyping often combined with others markers to assess the diversity of MTC strains. Spoligotyping appeared especially suitable as a simple and cheap tool to distinguish genotype families of this bacterium. Almost all international spoligotyping results have been submitted to an international database, SpolDB http://www.pasteur-guadeloupe.fr:8081/SITVITDemo/ that has already been updated 4 times [8]. Recent studies point to the potential use of CRISPR loci for molecular epidemiological studies of other pathogens [9], and an increased knowledge of these bacterial genomic structures is likely to foster the development of new high-throughput genotyping methods, either for studies on population structure or molecular epidemiology.

While spoligotyping does not differentiate *M. tuberculosis *isolates with the same level of discrimination as IS*6110*-RFLP (Restriction Fragment Length Polymorphism) [10] or MIRU-VNTR (Mycobacterial Interspersed Repetitive Units-Variable Number of Tandem Repeats) [11] it has several advantages [12]: i) it relies on a single PCR amplification which requires much less DNA quantities than IS*6110*-RFLP so that even smear-positive sputum samples can be directly analyzed, ii) up to 40 DNA samples can be analyzed within one day with the classical method and up to 186 with the novel microbead-based spoligotyping that has proven very reliable [13,14], iii) isolates with less than 6 copies of the IS*6110 *insertion element are better discriminated by spoligotyping than by IS*6110-*RFLP typing, and iv) spoligotyping patterns can distinguish between main lineages and sublineages within the *M. tuberculosis *complex (MTC) making them phylogenetically informative except in the rare cases where the exact same deletions (covering the exact same spacers) occurred by convergent evolution [9,14-17].

The classical spoligotyping described by Kamerbeek *et al*. revised by van Embden *et al*. is a robust method with an intra-laboratory reproducibility over 90% in well-trained laboratories [7,18]. However it can be affected by several issues: (i) it relies on the quality of the pre-prepared membrane and this has proven difficult even when commercialized; (ii) spoligotyping does not yield black or white results and reading is sometimes subjective; only results double checked by experienced staff seem reliable but this technically demanding procedure is not always implemented in specialized labs, (iii) the repeated use of the same membrane may be the cause of technical artifacts, (iv) data entry and classification are performed manually with an increased likelihood of errors during transcription [19]. All these issues might have impaired reliability in international genetic databases that have been compiled so far (SpolDB projects).

The transfer of the classical spoligotyping method into an advanced platform represents a technical progress because it allows to produce a numerical raw result format and a standardized signal/noise cut-off determination, ensuring both a high throughput and an high quality [13,14].

Hence, the aims of this study were to: (i) retrospectively assess the global quality of spoligotyping results that have been produced in various laboratories worldwide during a decade by comparing their data with those provided by the new, highly reliable system, (ii) give a feedback to the laboratories about their data production quality, (iii) solve uncertainties on samples in which centers are not confident, (iv) study if the DNA extraction procedure may influence membrane-based spoligotyping results, (v) assess the possible contribution of the microbead-based technique to an increased quality of services in molecular epidemiological studies. Altogether, this article identifies the main pitfalls in CRISPR data production, at a time when tuberculosis spoligotyping still inflates and is transferred towards other micro-organisms.

## Methods

### Oligonucleotides

The capture probes for microbead-based spoligotyping are identical to the ones from the membrane-based spoligotyping technique with minor modifications to correct some sequences from the original set [7,18]. All capture oligonucleotides (Eurogentec, Liège, Belgium) were modified at the 5' terminal amino group by a twelve carbon spacer linker to obtain the adequate free space between the microspheres and the oligonucleotides (increase of gyration radius).

### Spoligotyping PCR protocol

PCR (25 μl total) was performed for 20 cycles for CTAB DNA [7] and 25 cycles for thermolyzates using 2 μl of tested DNA.

### Hybridization

Was done in TMAC buffer at 52°C for 10 minutes as described before by Zhang *et al*. [14].

### Data analysis

For each spacer, hybridization signals were recorded as RFI (Relative Fluorescence Intensity) values and were transformed in a binary code (presence/absence) using a signal/noise cut-off value of 2 times the lower values' group. Octal codification and SpolDB4 lineage identification were assigned to each spoligotype. Spoligotypes generated by the membrane technique were compared to the Luminex generated spoligotypes, spoligotype by spoligotype and spacer by spacer. The first comparison allowed determining perfect matches (**pm**), *i.e*. obtaining exactly the same spoligotype by both methods (identical results for 43 over 43 spacers). The percentage of perfect matches in a center is calculated as the number of isolates with **pm **divided by the total number of isolates provided by the center. The second comparison dealing with individual datapoints (or spacers) was represented by the rate of difference (**rd**). This **rd **index indicates the number of discordant data points for the total number of data points analyzed per center: e.g. if 8 of 100 DNAs tested show discordant results in 2 spacers the **rd **value equals 8*2*100/(100*43) = 0.37%.

We also investigated if some spacers were more prone to errors (see additional file 1). Problematic spacers were defined as spacers having exhibited at least one discrepancy in a center. Consecutive problematic spacers are referred to as "blocks" of problematic spacers.

### Mycobacterial isolates, DNA extraction

DNA samples were provided by 11 centers that perform membrane-based spoligoyping as a routine procedure: i) Buenos Aires - Argentina (Servicio de Micobacterias, Instituto Nacional de Enfermedades Infecciosas, ANLIS "Carlos G. Malbran", Buenos Aires, Argentina); ii) Pisa-Italy (Università di Pisa. Dipartimento di Patologia Sperimentale, Biotecnologie Mediche, Infettivologia e Epidemiologia) and the Regional Reference Center for Mycobacteria, Firenze; iii) Bilthoven - The Netherlands (National Institute for Public Health and the Environment-RIVM); iv) Paris-France (Microbiology Laboratory, Hôpital Bichat-Claude Bernard, AP-HP); v) Madrid-Spain (Servicio de Microbiología Clínica y Enfermedades Infecciosas, Hospital General Universitario Gregorio Marañón); vi) Pointe-à-Pitre - Guadeloupe (TB and Mycobacteria Research Unit, Institut Pasteur de Guadeloupe); vii) Antananarivo-Madagascar (TB reference laboratory, Institut Pasteur de Madagascar); viii) Riyadh-Saudi Arabia (TB Research Unit, Comparative Medicine, King Faisal Specialist Hospital and Research Center); ix) Rio de Janeiro, Oswaldo Cruz Institute Laboratory of Molecular Biology applied to Mycobacteria, Brazil; x) Antwerp-Belgium, Mycobacteriology Unit, Prince Leopold Institute of Tropical Medicine, and xi) Brussels-Belgium, Scientific Institute of Public Health, National Reference Centre of Tuberculosis and Mycobacteria.

These selected strains have a worldwide origin and represent a wide range of MTC genotypes. DNA was extracted using a thermolyzate or a cetyl-trimethyl-ammonium bromide (CTAB) procedure. DNA samples fulfilled the following criterion: samples representative of hard to interpret spoligotypes and samples representative of the work flow, so we could evaluate the benefits of a new platform regarding the classical one on these samples. Most centers had performed spoligotyping using commercial membranes (Ocimum, Hyderabad, India) (except for the laboratories in Institut Pasteur of Guadeloupe, Institut Pasteur of Madagascar, and the University of Pisa). Samples were analyzed in a blind way which means that we did not know the profiles from the membrane-based spoligotyping until we had everything processed by the microbead spoligotyping. We named the centers from 1 to 11 for convenience ranked according to their spoligotyping quality, so this numbering is not correlated with the order in which they are described by name to respect confidentiality. The number of samples included in this study by Center are as follows: 1 (49 samples), 2 (95 samples), 3 (98 samples), 4 (97 samples), 5 (87 samples), 6 (82 samples), 7 (120 samples), 8 (70 samples), 9 (59 samples), 10 (85 samples) and 11 (85 samples).

### Statistical analysis

Homogeneity "Mann-Whitney" non-parametric U test was performed in an Excel sheet and "Spearman rank correlation" test (with Rho and p calculations) was performed using the online tool at http://www.u707.jussieu.fr/biostatgv. df = degrees of freedom, relating the number of independent observations used to compute the statistical parameters.

## Results

**Figure 1:**
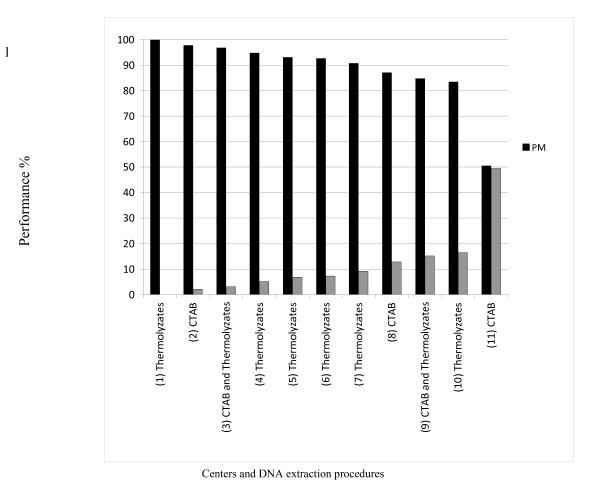
**Relative quality of the classical membrane-based spoligotyping by center**. One hundred percent (100% performance) quality is inferred when obtaining complete (identical) concordance, i.e. 43/43 spacers, with the high-throughput based spoligotyping for every isolate. The centers were numbered according to their spoligotyping quality and these numbers are shown between parentheses. DNA extraction procedure is also mentioned for each center.

Eight centers using commercial membranes and three using in-house made membranes were included in the study, totaling 927 isolates. We performed the microbead-based spoligotyping on Luminex on the same set of DNA samples as a new standard, even though it is clear that only sequencing would provide full reference information on spacer sequences and genomic structure of the Direct Repeat locus, especially in case of doubtful hybridization results for some spacers [13,14,18]. Centers were ranked from 1 to 11 according to their performance. The seven best centers reached over 90% of **p**erfect **m**atch (pm = exactly the same spoligotype pattern over the 43 spacers), three centers obtained between 87 and 84% concordance and one laboratory only 51% (Figure 1). However, this specific center had a high level of errors at a single spacer, and when ignoring the errors at that spacer, it reached 88% of **pm **(data not shown).

Participating centers used either Cetyl-Trimethyl-Ammonium-Bromide (CTAB) extracted or heat-shock extracted (thermolyzates) DNA, depending on the type of studies they usually use the DNA for: the CTAB-based extraction method is preferred for molecular analyses demanding high purity (e.g. IS*6110*-RFLP for which CTAB is mandatory), whereas thermolyzates are used in other cases due to its swiftness and ease of implementation. Surprisingly, the best performing center (Center 1) used only thermolyzate DNA, and Center 11 used only CTAB DNA (Figure 1). Altogether, centers that used the thermolyzate DNA extraction procedure performed as well as centers using the CTAB procedure (n_CTAB _= 3, n_Thermolyzates _= 6; Mann Whitney test: = U = 6; p = 0.374).

**Figure 2:**
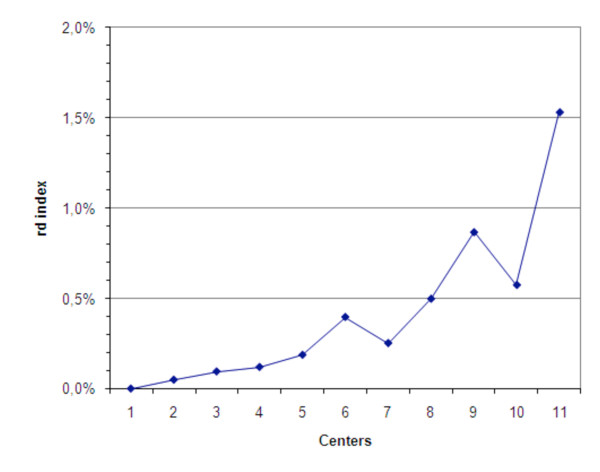
**Datapoints discrepancies per center**. Datapoints consist in every spacer of each spoligotype pattern. The percentage of differences among all datapoints is referred to as rd for rate of difference.

Each spoligotype is a string of 43 characters so that in total 39,861 data points were analyzed in this study. Our comparative results showed 157 (0.39 %) data point discrepancies. We defined the rate of difference (**rd**) index as the number of mismatches divided by the total number of analyzed data points in each center (see material and methods). All centers except one exhibited a "**rd**" below 1% (Figure 2). In addition, when ignoring the errors due to the recurrently problematic spacers in most centers, **rd **dropped to 0.38%. As expected, **rd **was correlated to the rank of centers (Spearman rank correlation test Rho = 0.99; df = 9; p < 0.001): centers having the highest numbers of erroneous spoligotype patterns also had the highest number of individual spacers errors. Still, specific centers had a relative low **rd **as expected from their **pm**: centers 6 and 9 exhibited slightly higher global quality (as measured by the **pm**) than centers 7 and 10 respectively (Figure 1), however, they had a higher amount of individual discrepancies (as measured by their **rd**, Figure 2). This indicates that, in these centers, the mismatches were often belonging to the same spoligotype patterns. No statistical difference in the **pm **relatively to the use of commercial or home-made membranes was observed (n_Home _= 3; n_Commercial _= 8; Mann Whitney test: U = 10; p = 0.387 i.e. non significant).

**Figure 3:**
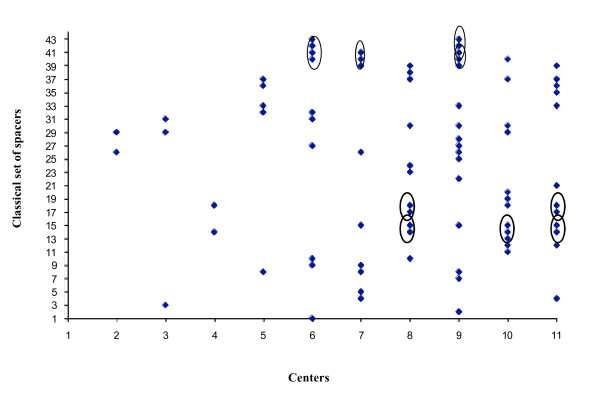
**Problematic spacers by center as derived from the comparison of spoligotypes generated by both methods**. Blocks of adjacent problematic spacers are circled.

**Figure 4:**
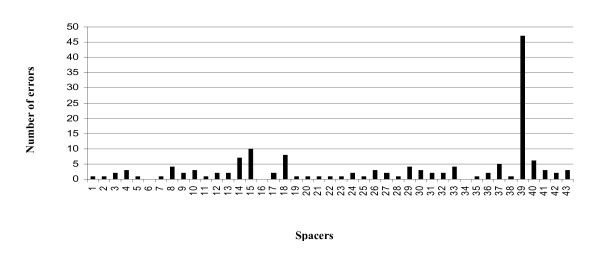
**Number of datapoints genotyping errors per spacer**.

We also looked for potentially problematic spacers creating systematic mismatches in multiple centers. Spacers that were wrongly identified by the classical spoligotyping varied among centers (Figure 3). They occasionally were located adjacent one to another and part of the corresponding blocks were shared by two to three centers (see circles, for instance for spacers 40 and 41, in Figure 3). However, most problematic spacers were not generally shared. The spacer that most frequently introduced errors was spacer 39 (Figure 4) but it was not detected among 42 isolates in one specific center, likely due to a membrane production problem. When ignoring these samples, spacers most prone to introducing errors were spacers 15, 18, 14 and 40; and to a lesser extent spacers 37, 8, 26, 29, 30, 33 and 41 (Figure 4).

## Discussion

We show in this study that different centers performing membrane-based spoligotyping exhibit different inter laboratory reproducibility rates ranging from 51 to 100% and most of them reaching more than 90% of quality as represented by their **pm**. One center had a specific detection problem of spacer 39. Ignoring the errors associated with this spacer, it reached a **pm **as high as the other centers. The choice of the sample was initially meant to include problematic specimens as well as sample representative of the work flow, however a separate statistical analysis between the random work flow and hard-to-type samples was not feasible here (low effective size), hence the discrepancies reported here are upper limits and could have been lower if only random selection had occurred.

The reasons why spoligotyping errors occur can be diverse. First, DNA quality associated to the extraction procedure could be critical. In this study, however, the DNA extraction procedure did not influence the performance of the membrane-based spoligotyping. Nevertheless, we have to keep in mind that we only tested CTAB and thermolyzates and other extraction procedures were not tested. Some centers repeatedly introduced genotyping errors at multiple spacers in the same samples. Some samples may have been of inadequate quality for all spacers to be sufficiently amplified. However, this occurred both for centers using CTAB and thermolyzate DNA, confirming that factors other than extraction procedure, such as the amount of extracted DNA, inclusion of culture medium when preparing bacterial suspensions or possible culture contaminations, may have influenced PCR quality and hence modified the spoligotypes of some samples.

Regarding artisanal process of membrane production, an adequate quantity of specific probe concentrations on the membrane could sometimes be defective which would lead to the same spacer being recurrently problematic in the same center. Also, commercial membranes might experience some spacer problems so internal quality control must be done by each lab. Indeed, we have observed that in Center 11, spacer 39 was affecting the results of 42 strains. However, in other centers, such problems were not observed. We advise positive and negative controls to be included at randomly chosen positions to detect such possible membrane production problems; centers producing their own membranes should also check their production adequately before use.

Third, operator-dependent washing problems could occur on any part of the membrane so that any spacer could be wrongly scored for some isolates. Insufficient washing can lead to false positive detection of a spacer. As an example, spacers 14 or 37 were detected, although they proved to be absent. In contrast, excessive washing will level the signals of several probes down. All centers used their membranes between 8 to 14 times which is under the limit advised by membranes' manufacturer (up to 20 times). However, this might still be too high. We advise users to always record the results with positive and negative controls included at randomly chosen positions to detect the membrane fall down in quality, and possible insufficient washes.

Fourth, there can be intrinsic problem due to the CRISPR structure in MTC: some spacers constantly provide weaker signals that make the distinction between positive and negative values more difficult. For instance, DVR next to spacer 15 (DVR26) harbors a deletion of 4 nucleotides at its 5' end as shown in highly diverse strains [18]. Consequently, PCR primers do not hybridize properly around spacer 15 which likely leads to a lower amplification level of this spacer.

The high-throughput spoligotyping method has the advantage of being more sensitive to detect these slight variations, given the 3D nature of the immobilized probes, which provides more surface to attach more PCR products. Moreover, unspecific hybridization is removed more easily with the microbead technology, making the detection of spacer 15 possible in any sample. Contrarily, in our study, spacer 40 was detected several times by membrane-based spoligotyping, but not by the microbead-based technique (false positive on membrane).

Fifth, the hybridization reading from the film, and the transfer of this information to a digital format have to be handled manually in the membrane-based spoligotyping increasing the transcription error risk; the best procedure to reduce this risk is to have the results read in duplicate by two independent readers, to check reading mistakes and to solve potential discrepancies.

Reading errors are suppressed in the high-throughput method due to an in-house designed automatized routine, which already provides a digital file with a suggested and objective interpretation according to predefined thresholds (cut-off).

As the distribution of wrongly typed spacers is random (see additional file 1), low quality reading and transfer of results may be the major problem in the classical spoligotyping. This can be particularly misleading when errors concern spacers that are used to classify strains into genotype level. For instance, spacer 18 has been proposed to be highly informative to identify the MTC "X" clade strains, and spacer 40 to define the T2 subclade [8,20].

## Conclusions

CRISPR region genotyping in MTC, referred to as spoligotyping, revealed very useful for first line screening of epidemiological analyses and remains a robust and widely accepted genotyping method [21,15]. Since the technical improvement of microbead-based versus membrane-based spoligotyping method was previously demonstrated in two independent settings, we took microbead-based method as a new standard to study retrospectively the quality of membrane-based spoligotyping results in 11 centers [13,14]. However, sequencing will remain the unique reference method for CRISPR genomic structure in case of doubts. The performance of the membrane-based spoligotyping was shown not to be influenced by the DNA extraction procedure. Overall, a relative good performance was obtained in most centers. Even if globally reliable, we report here that membrane-based spoligotyping suffers some practical limitations which are overcome when switching to the microbead-based format. This format remains more expensive even though the higher reagents cost is partly balanced by decreased add-ons (high-throughput) and interpretation time.

A single center out of eleven had a systematic problem of membrane quality that was overlooked (non reactivity of spacer 39). This issue was due to a problem in membrane production. Other centers yielded errors slightly more frequently at spacer 15, that is known to be less amplified because of its neighboring modified CRISPR sequence around this spacer. In contrast, the increased sensitivity of the high-throughput method was validated by the proper detection of this spacer [13,14]. Other genotyping errors by classical membrane-based spoligotyping most likely are due to variation in interpretation of weaker spots and/or transferring of data from the film to a digital file processes.

The high-throughput microbead-based method was shown to have a better sensitivity due to proper detection of some spacers that can be misinterpreted by the classical method, and a lower error rate due to automated interpretation and transcription. A new 68 spacers microbead-based spoligotyping format provides an increase in discriminatory power for Principal Genetic Group I clinical isolates, a feature that could be especially useful in South-East Asia where the East-African Indian (EAI) clade predominates [14].

The launch of a new Luminex device (MagPix), with a price drop, yet lower-plex, molecular diagnostics instrument (50Plex) that would be interesting for fields labs (e.g. without requirement of air conditioning) could facilitate the spreading of spoligotyping; however this will only happen if routine utilization (through a concomitant drop in reagents prices) is done within acceptable cost limits for public health and an improved quality of service.

## Competing interests

The authors declare that they have no competing interests.

## Financial Competing interests

EA, JZ, GR and CS declare that they never received any direct financial support by the Luminex Corp. or Luminex BV companies as well as no staff support was provided by Luminex Corp. nor Luminex BV to perform this study.

## Authors' contributions

EA and JZ performed and analyzed the microbead-based spoligotypes, VR, KK, RR, LR, HMG, AR, PS, MFD, KS, MH, VR, MH, DGV, SAAN, NR, CG, ET, DvS, participated in sample recruitment and performed membrane-based spoligotyping in their respective laboratories, GR performed the Statistical analyses, CS conceived and coordinated the study, EA, GR and CS wrote the manuscript. LR, VR, DvS, DGV contributed to the manuscript revision and all authors approved the final version of the manuscript.

## Pre-publication history

The pre-publication history for this paper can be accessed here:

http://www.biomedcentral.com/1471-2334/11/110/prepub

## Supplementary Material

Additional file 1 **Center's distribution of problematic spacers**. This file highlights the problematic spacers shared by several centers (lines). Click here for file
