# Supplementary material for: The use of microbead-based spoligotyping for Mycobacterium tuberculosis complex to evaluate the quality of the conventional method: Providing guidelines for Quality Assurance when working on membranes
Source: BMC Infect Dis. 2011 Apr 28;11:110. doi: 10.1186/1471-2334-11-110 (PMC3107175; doi:10.1186/1471-2334-11-110)

Supplementary File 1.

Center's distribution of problematic spacers highlighting those shared by several centers (lines).

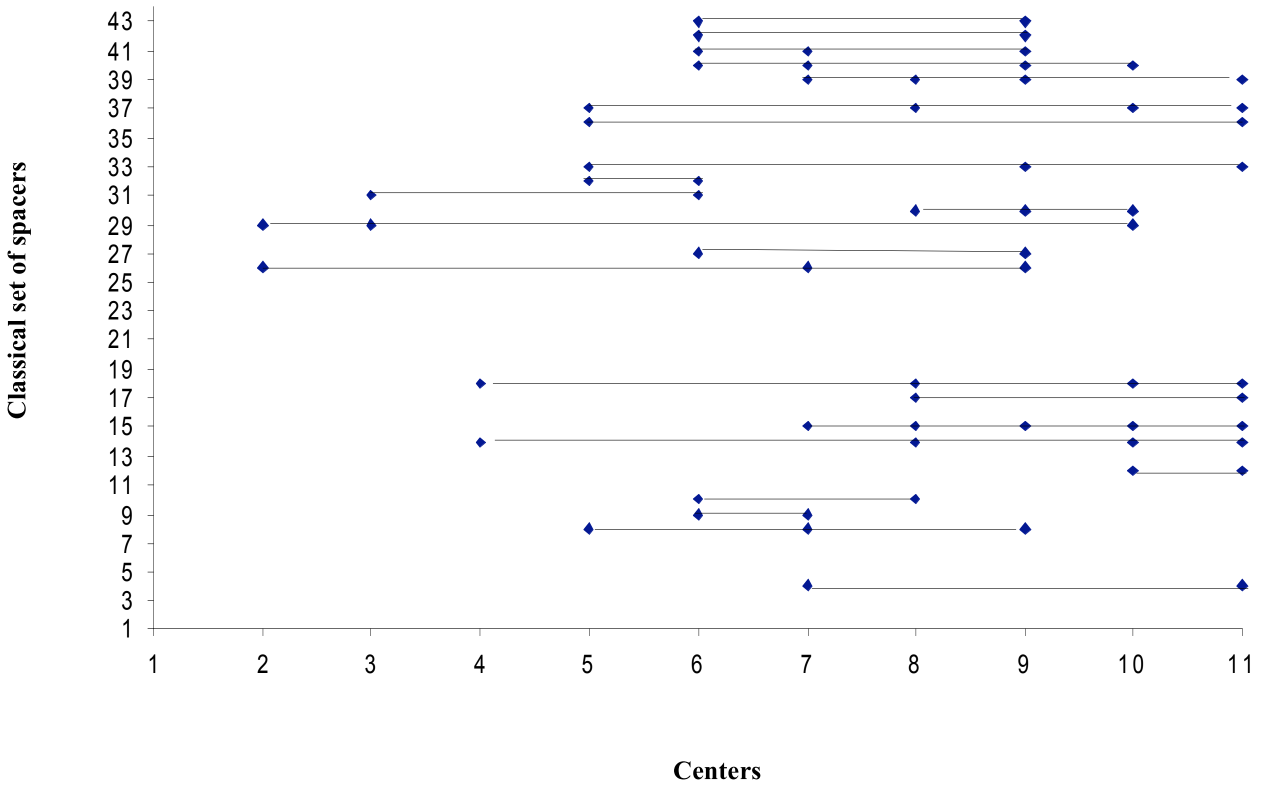

Supplement: Additional file 1 — Center's distribution of problematic spacers. This file highlights the problematic spacers shared by several centers (lines). [file 1471-2334-11-110-S1.PDF]
